# VPS13 and bridge-like lipid transporters, mechanisms, and mysteries

**DOI:** 10.3389/fnins.2025.1534061

**Published:** 2025-04-28

**Authors:** Laura Elizabeth Swan

**Affiliations:** Department of Biochemistry, Cell and Systems Biology, University of Liverpool, Liverpool, United Kingdom

**Keywords:** BLTP, VPS13, ATG2, scramblase activity, lipid transfer activity, membrane contacts

## Abstract

Bridge-like lipid transporters (BLTPs) have recently been revealed as key regulators of intraorganellar lipid trafficking, with their loss being associated with defective synaptic signalling and congenital neurological diseases. This group consists of five protein subfamilies [BLTP1-3, autophagy-related 2 (ATG2), and vacuolar protein sorting 13 (VPS13)], which mediate minimally selective lipid transfer between cellular membranes. Deceptively simple in both structure and presumed function, this review addresses open questions as to how bridge-like transporters work, the functional consequences of bulk lipid transfer on cellular signalling, and summarises some recent studies that have shed light on the surprising level of regulation and specificity found in this family of transporters.

## Introduction

For several decades, subcellular organelles were considered distinct entities, whose membrane lipid content could only be changed by the action of organelle-targeted enzymes, by subcellular sorting performed by adaptor proteins bending and sorting lipids into specific geometries to be scissioned and trafficked away from its parent organelle, or by admixture of organelle membranes via mechanisms such as SNARE-mediated fusion. Excitingly, recent decades have revealed the existence of non-vesicular lipid transfer by specialised transporters at proteinaceous organelle-to-organelle junctions (membrane contacts) whereby selected membrane lipids are exchanged across a narrow organelle-to-organelle gap.

In the last few years, genetic studies combined with structural insight from protein modelling (Braschi et al., 2022; Neuman et al., 2022a) revealed a significant group of structurally similar proteins, where the lipid transporter, rather than fostering an exchange mechanism, forms a physical bridge that spans the gap between a donor and a receiver organelle. These bridge-like transfer proteins (BLTPs) are formed by multiple repeats of one motif: a repeating beta groove (RBG) (Neuman et al., 2022a), which forms an extremely long semi-open tube with a hydrophobic interior (Li et al., 2020). These repeats and the tube they form are half-open to solvent, whereby lipids are conducted from one end to the other of the organelle gap by a single protein. This gap can span as much as ~30 nm between organelles and is known to have critical roles in establishing membrane function. Our current understanding of the function of BLTPs is relatively crude: they are thought to be comparatively unselective in the lipids they transport and to act essentially as a ‘firehose’ delivering phospholipids to their target membranes. However, as our understanding of these mechanisms develops, researchers have found a surprising amount of subtlety as to how each of the five members of the BLTP family functions, and how lipid transfer via each of these transporters leads to signalling and trafficking deficits in human patients and model organisms.

Understanding the properties, regulation, and function of this class of proteins will provide significant insight into how organelles maintain their identity, along with transmembrane cargo and adaptors, while allowing response to physiological changes. This review discusses some of the key open questions concerning the nature and function of these proteins and presents current evidence as to how this family of proteins works to drive organelle function.

### The structure of bridge-like transporters

The group of bridge-like transporters (BLTPs) form a small, but well-conserved (Table 1) group of five protein groups(Braschi et al., 2022; Neuman et al., 2022a) BLTP1-3, autophagy-related 2 (ATG2) and vacuolar protein sorting 13 (VPS13), and their paralogues. The group shares some common features: an N-terminal Chorein domain [a scoop-shaped domain with a hydrophobic interior, whose evolutionary origins may extend as far back as a common ancestry with bacteria (Neuman et al., 2022a; Levine, 2019)], which directly funnels into the interior of anywhere from 6 to 17 repeats of the Repeating Beta Groove (RBG) motif (Neuman et al., 2022a; Levine, 2022). The RBG motif is a five antiparallel beta-stranded repeat that curves into a U or ‘taco’ shape and terminates in an unstructured loop, which when repeated, spans the distance between donor and acceptor membrane. BLTPs show extensive and conserved cytosolic loops and patches that decorate the cytosolic length of the tube formed by the RBG repeats (Dall’Armellina et al., 2023), but few specific interactors of these patches are known. At both the N- and C-termini of these proteins, which form the interfaces with donor and acceptor membranes, the proteins become more specialised among individual family members. Nearly every member of this family has been associated with human disease (Ugur et al., 2020; see Table 2), highlighting the important physiological role that this form of lipid transport plays.

**Table 1:** Known orthologues of BLTP proteins (alternative names for the same gene in brackets).

*H. sapiens*	*D. melanogaster*	*C. elegans*	*S. cerevisiae*	*D. discoideum*	*A. thaliana*
BLTP1 (KIAA1109)	Tweek	lpd-3	CSF1	DDB_G0289829	
BLTP2 (KIAA0100)	Hobbit	bltp-2	Fmp27 (HOB1), HOB2		SABRE, KINKY POLLEN
BLTP3A (UHRF1BP1) BLTP3B (SHIP164/UHRF1BP1L)	CG34126	C44H4.4		DDB_G0279089	AT3G20720 (Q84R14)
ATG2A, ATG2B	Atg2	atg-2	ATG2	Atg2 (DDB_G0277419) DDB_G0282057	ATG2 (Wang et al., 2011)
VPS13A (ChAc) VPS13B, VPS13C VPS13D	Vps13, Vps13B, Vps13D	VPS-13A (T08g11) VPS-13D (C25H3.11) (Levine, 2022)	VPS13	VPS13A VPS13B VPS13C (TipC), VPS13D VPS13E VPS13F (Leiba et al., 2017)	AtVPS13S (Shrubby) AtVPS13M1 AtVPS13M2(Velayos-Baeza et al., 2004) AtVPS13X (Levine, 2022)

**Table 2:** Diseases associated with BLTPs, recruitment factors for N terminal and C terminal domains of BLTPs with specific subdomains if known.

Protein	Disease	N-terminal membrane (except chorein domain)	C-terminal membrane
BLTP1 (KIAA1109)	Alkuraya-Kucinskas Syndrome (Alazami et al., 2015; Gueneau et al., 2018) (MIM 617822)	Transmembrane helix (Kang et al., 2024a) spigot (C1orf43) via chorein and N terminal RBGs (Kang et al., 2024a) ER	Polybasic patch (expected to bind Phosphoinositide lipids) (Wang et al., 2022) Plasma membrane
BLTP2 (KIAA0100)		transmembrane helix (Neuman et al., 2022b) ER	Phosphoinositide lipids (PI, PIP, PIP2, PIP3) (Neuman et al., 2022b) PI4P (Dai et al., 2025) FAM102A/B *via* helical binding domain (Dai et al., 2025) Amphiphysin2 *via* RXP SH3 binding motif (Dai et al., 2025) Plasma membrane
BLTP3A (UHRF1BP1)	Systemic lupus erythematosus (Wen et al., 2020)	**rab7** *via* RBG2 (Hanna et al., 2025) late endosome/lysosome	***Rab45*** (Hanna et al., 2022) Centrosome ? via RBG6 (Hanna et al., 2025) VAMP7-positive vesicles LC3, GABARAP-*via* LIR motif (Hanna et al., 2025) Stressed/damaged lysosome
BLTP3B (SHIP164/UHRF1BP1L)	Parkinson’s disease (Jansen et al., 2017)	***Rab5*** (Gillingham et al., 2019) Early endosomes ***RhoBTB3*** (Wang et al., 2024) Golgi	syntaxin 6 (Otto et al., 2010) *via* LxxYY motif (Hanna et al., 2022) Early/recycling endosomes DyneinLL1/2 (Hanna et al., 2022) via C-terminal peptide ***Rab45*** (Hanna et al., 2022) Centrosome VPS26B (Wang et al., 2024) M6PR-positive endosome (not retromer)
ATG2A(BLTP4A) ATG2B (BLTP4B)	ATG2B: Familial susceptibility to myeloproliferative neoplasms (MIM: 616604) (Saliba et al., 2015)	**ATG9** (Wang et al., 2024) **TMEM41B** **VMP1** (Ghanbarpour et al., 2021) ER	Liprin-like domain (Kotani et al., 2018; Van Vliet et al., 2022) GABARAP/GABARAPL1/LC3A via LIR motif (Bozic et al., 2020) WIPI4 (Maeda et al., 2019; Chowdhury et al., 2018) via YFS motif (Zheng et al., 2017) **ATG9** (Wang et al., 2024; Van Vliet et al., 2022) Phosphoinositide lipids (PI3P) (Kaminska et al., 2016) Phagophore TOM40 via MAM localisation domain (MLD) (Tang et al., 2019) Mitochondria associated ER membrane/phagophore
VPS13A (BLTP5A)	Chorea-acanthocytosis (MIM: 200150) (Rampoldi et al., 2001)	VAPA/B via FFAT-motif (Yeshaw et al., 2019) ER	**XK** (Adlakha et al., 2022) via PH domain (Guillen-Samander et al., 2022) Plasma membrane ? via PH domain (Guillen-Samander et al., 2022) mitochondria SNX5 via VAB (Tornero-Ecija et al., 2023) ***rab7*** (Munoz-Braceras et al., 2019) late endosome/lysosome Lipid membranes via liprin-like domain/amphipathic helices (Kumar et al., 2018)
VPS13B (BLTP5B)	Cohen syndrome (MIM: 216550) (Kolehmainen et al., 2003)	FAM177A1 (Ugur et al., 2024) via? Golgi	Sec23IP via VAB (Du et al., 2024) ER-exit site (ERES) Phosphoinositide lipids (PI4P) (Du et al., 2024) via PH (PI3P) (Koike and Jahn, 2019) via? ***Rab14*** (Pietra et al., 2013) endosomes Syntaxin 6 and syntaxin 13 together (Koike and Jahn, 2019) Transferrin receptor-positive early/recycling endosomes ***rab6A/B/C*** (Seifert et al., 2015) Golgi
VPS13C (BLTP5C)	Autosomal recessive early onset Parkinson’s Disease 23 (Lesage et al., 2016; Jansen et al., 2017) (MIM: 616840) Dementia with Lewy bodies (Smolders et al., 2021) (MIM:127750)	VAPA/B via FFAT-motif ER	***pT73-rab10*** (Schrӧder et al., 2024) lysosomes ***rab7*** via VAB (Hancock-Cerutti et al., 2022) late endosomes/lysosomes liprin-like domain/amphipathic helices (Kumar et al., 2018) phosphoinositide lipids
VPS13D (BLTP5D)	Autosomal recessive Spinocerebellar Ataxia 4 (Seong et al., 2018; Gauthier et al., 2018) (MIM: 607317)	? via N terminal region (Wang et al., 2021) mitochondria VAPB via pFFAT-motif (Guillen-Samander et al., 2021) ER	***Mitofusin 2*** (Shen et al., 2021) ***Miro1/2*** via VAB (Guillen-Samander et al., 2021) Mitochondria/peroxisome liprin-like domain (Wang et al., 2021) TSG101 via VAB domain (Wang et al., 2021) lipid droplets K63-linked ubiquitin via UBA domain (Anding et al., 2018) p97/VCP via UBA and VAB domains (Du et al., 2021)
yeast VPS13		LIR motif ER	**MCP1** to VAB domain via PXXP motif (Adlakha et al., 2022; Bean et al., 2018; Gonzalez Montoro et al., 2018) mitochondria spo71 to VAB domain via PXXP motif (Bean et al., 2018; Park et al., 2013) prospore membrane ypt35 to VAB domain via PXXP motif (Bean et al., 2018) endosome/vacuole ***Arf1*** via PH domain (Kolakowski et al., 2021) Golgi phosphoinositide lipids (PI3P) (Rzepnikowska et al., 2017; De et al., 2017) [PI(4,5)P_2_] (Kolakowski et al., 2021)

**Bold underline** , proteins with scramblase activity. ***Bold italics***, small GTPases or dynamin-like GTPases. Interactors that have not been definitively associated with a direct physical interaction with the N or C terminus of BLTPs are omitted or indicated with a question mark. Red, organelle recruitment defined by the specified interactor(s). Please note that other organelle junctions sporting BLTPs have been found where the interaction that defines the recruitment is not known. LIR, LC3 interacting region.

**Figure 1:**
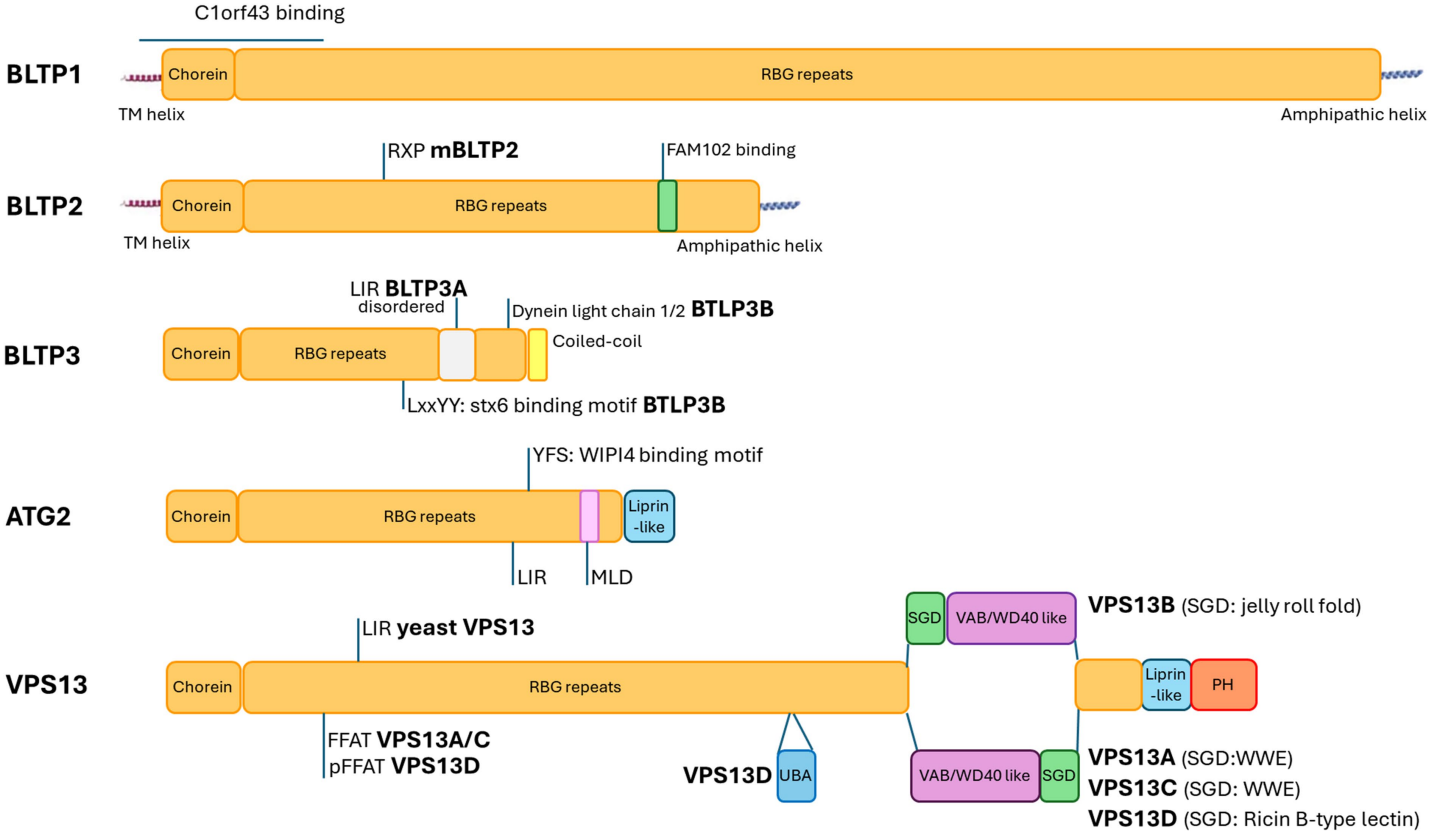
Cartoon of domain structure of BLTPs, with some specific interaction surfaces labelled. MLD: MAM localisation domain, UBA: ubiquitin-associated; SGD: small globular domain, VAB: VPS13 adaptor-binding domain, PH: pleckstrin homology domain, LIR: LC3 interacting motif; RXP: SH3 binding peptide motif; (p)FFAT: (phospho) Two phenylalanines in an acidic tract peptide motif.

**Figure 2:**
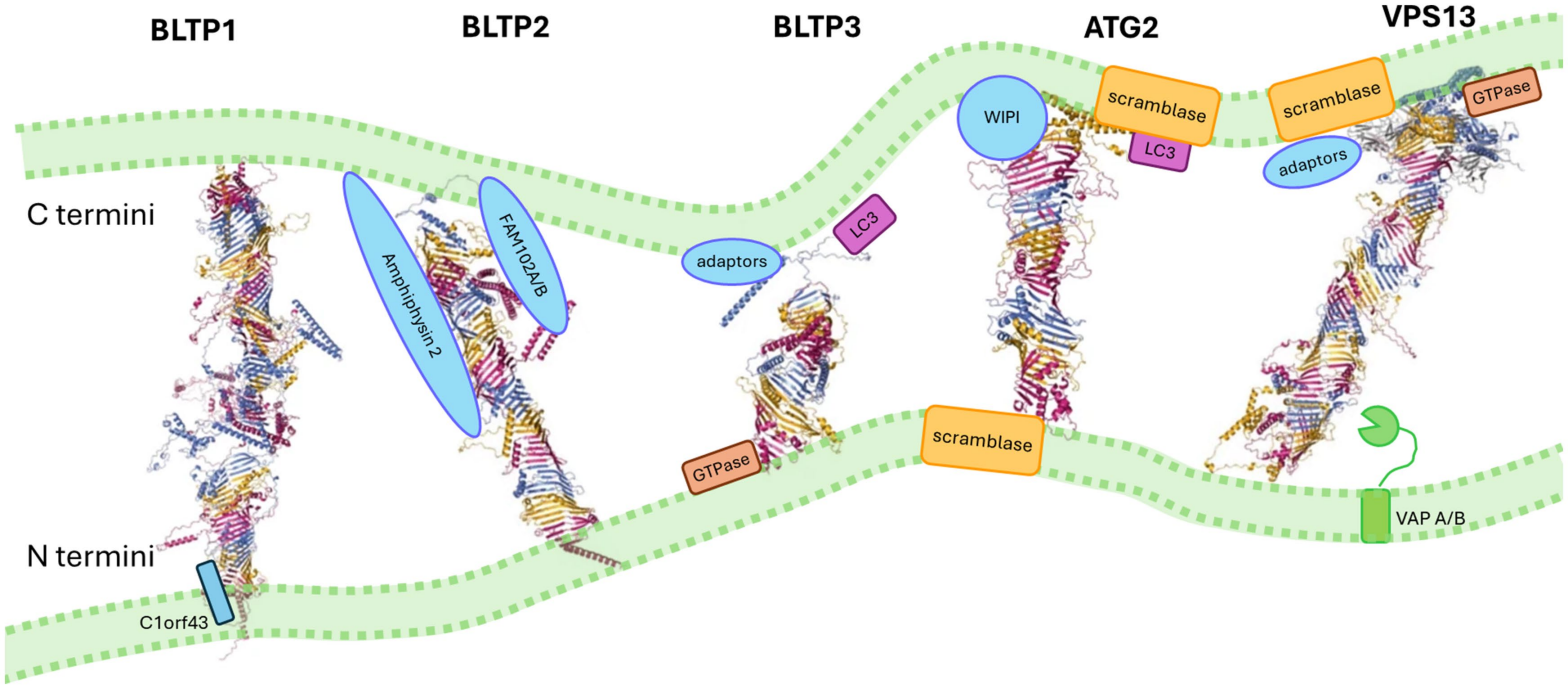
Representative structures of BLTPs. Models of generic BLTP family members taken from Braschi et al. (2022), where the authors coloured each RBG repeat in magenta, gold, and teal sequentially (https://creativecommons.org/licenses/by/4.0/). Proteins are aligned, so the N terminus interacts with the lower of the two generic membranes. When orthologues or paralogues bind interactors of a similar functional type, then the type of protein, rather than the specific interactor, is named.

Very loosely, the BLTP family of proteins can be split into two groups: BLTP1-3, which do not have extensive C-terminal interfaces with adaptor proteins, and ATG2/VPS13 families (designated BLTP4/BLTP5 respectively), which have extensive C-terminal specialisations that form a platform to recruit multiple adaptor factors on their target membrane. For a cartoon of the domain structures of BLTP family proteins, see Figure 1. A model of their association with membranes is presented in Figure 2.

BLTP1 and BLTP2 have a N-terminal transmembrane helix that anchors the bridge (Kang et al., 2024a) to a prospective donor membrane, whereas the N-terminus of the group proteins formed by ATG2, VPS13 loosely anchor to their N-terminal membranes either by interacting with tethering proteins via peptide motifs or by weak interaction with membranes through their chorein domain (Levine, 2022; see Table 2). The C-terminus of the ATG2/VPS13 grouping is more highly specialised and provides a surface for interactions with adaptor proteins on the acceptor or target membrane. Interestingly, the recruiting motifs for BLTPs are often either small GTPases and/or phosphoinositide lipids (see Table 2), which suggest that recruitment may be dynamic and tied to signalling. In fact, in studies so far, very often the recruitment of BLTPs to specific junctions depends on the level of expression of their putative interaction partners. BLTP3 proteins are even less specialised at their N and C termini (having neither transmembrane helix nor specialised C terminal domains) and may be recruited by interactions with small GTPases(Hanna et al., 2025; Hanna et al., 2022; Gillingham et al., 2019; Wang J. et al., 2024).

In BLTP1-2, C-terminal specialisations are relatively simple: The C terminus is denoted by a single-helical segment, which has been demonstrated to interact with membrane phosphoinositide lipids (Wang et al., 2022; Neuman et al., 2022b), while peptide motifs in BLTP2 contact membrane adaptor proteins (Dai et al., 2025). In the ATG2/VPS13 grouping, this is more complicated: For ATG2A/B, the C-terminus sports an amphipathic helical bundle and an interface to interact with the WDR (WD40 repeat) family of proteins, such as WIPI4 (Chowdhury et al., 2018), which itself binds the phosphoinositide lipid PI3P. VPS13 family proteins are even further specialised with a cluster of well-conserved structures at the C terminus: a small globular domain [of varying fold in the different VPS13 family members, see Figure 1 (Levine, 2022; Dall’Armellina et al., 2023)], a ‘collar’ of Vps13 Adaptor-Binding (VAB) domain repeats forming a similar fold to WDR40 repeats at the end of the tube formed by the RBG repeats, a liprin-like amphipathic helical domain which, models suggest, contacts and disturbs the target membrane outer leaflet (Dall’Armellina et al., 2023), and PH (pleckstrin homology) domains all decorating the C-terminal region. In VPS13 family proteins, the PH domain also interacts with phosphoinositide lipids (Rzepnikowska et al., 2017; De et al., 2017; John Peter et al., 2017).

In VPS13/ATG2 family proteins, this extra series of C-terminal domains appears to specify an interacting surface for proteins on the adaptor end of the bridge to recruit BLTPs to their target membrane, allowing dynamic refinement of the recruitment of bridge-like transporters to their target membranes. Ultimately, however, the C-terminal specialisations of these proteins are not completely necessary for function, as loss of ATG2 in mammalian cells can be rescued by strong overexpression of the N-terminal portion of ATG2 alone (Valverde et al., 2019). This suggests that the minimum requirement is the ability to retrieve lipids from the donor membrane and some kind of channel to carry the solubilised lipid, which can, if necessary, randomly incorporate into its acceptor membrane.

### A brief overview of BLTP functions

Functionally, BLTPs are known to play a crucial role in autophagy, particularly in facilitating the rapid expansion of the phagophore membrane through proteins such as the well-studied ATG2 proteins (recently reviewed here; Vargas Duarte and Reggiori, 2023; Choi et al., 2024) although other BLTPs, such as VPS13 family members (Anding et al., 2018; Munoz-Braceras et al., 2015; Lei et al., 2022), have been found to contribute to autophagy in a number of model systems, including yeast where VPS13 and ATG2 act redundantly(Dabrowski et al., 2023). Intriguingly, BLTP3A, while not recruited by autophagy (Hanna et al., 2025), has recently been implicated in a similar process: Conjugation of Atg8 to Single Membranes (CASM) reviewed here (Durgan and Florey, 2022), a non-canonical process where single membranes rather than phagophores recruit elements of the autophagy machinery, dependent on the activity of the lysosomal V-ATPase. The other family members are also responsible for large membrane reorganisations: BLTP1 is required for phagocytosis in macrophages (Jeng et al., 2019) and astrocytes (Kang et al., 2023).

Beyond autophagy, members of this family are implicated in numerous essential cellular processes, such as mitochondrial homeostasis (Jansen et al., 2017; Yeshaw et al., 2019; Munoz-Braceras et al., 2019; Lesage et al., 2016; Anding et al., 2018; Nagata et al., 2018; Pandey et al., 2024), cell polarity (Pietra et al., 2013), organelle sorting (Otto et al., 2010), cargo trafficking (Koike and Jahn, 2019; Seifert et al., 2015; Neuman and Bashirullah, 2018), formation of lipid droplets (Wang et al., 2022; Yeshaw et al., 2019; Kumar et al., 2018; Wang et al., 2021), synaptic vesicle endocytosis (Verstreken et al., 2009), phagocytosis (Kang et al., 2023), ciliogenesis(Valverde et al., 2019), coordination of signalling cascades on specific organelle membranes (Parolek and Burd, 2024; Khuong et al., 2010), regulation of organismal growth (Neuman and Bashirullah, 2018; Tokai et al., 2000), and the dynamic modulation of membrane properties (Khuong et al., 2010; Banerjee et al., 2024).

Frequently, BLPTs have been associated with inherited disorders (see Table 2), typically linked to recessively inherited disorders of the nervous system, such as the severe neurodevelopmental disorder, Alkuraya-Kučinskas syndrome (BLTP1), a commonly perinatal lethal disorder affecting multiple systems, particularly brain development, where surviving patients suffer seizures, cardiac and renal symptoms, and some degree of intellectual disability (Gueneau et al., 2018). There are four VPS13 family members in humans, each associated with a neurodevelopmental disorder: VPS13A mutations cause Choreoacanthocytosis, a progressive disorder in which patients develop chorea, or a Parkinson’s disease-like dystonia without chorea (Monfrini et al., 2023), behavioural changes, and cognitive decline, marked by star-shaped red blood cells (acanthocytes) (Peikert et al., 1993); VPS13B is associated with Cohen syndrome, a disorder of developmental delay, intellectual disability, truncal obesity, progressive retinal dystrophy, and microcephaly; VPS13C is mutated in a young-adult onset form of Parkinson’s disease, showing rapidly progressive degradation of dopaminergic neurons, with accompanying cognitive and motor decline, or in other cases, dementia with Lewy bodies (neurodegeneration characterised by parkinsonism, episodic changes in cognitive function, hallucination), while VPS13D is implicated in spinocerebellar ataxia, which manifests as developmental delay, and perinatal to adult-onset seizures and movement disorders such as spastic ataxia, spastic paraplegia, and chorea. BLTP3B was also found to be mutated in an early-onset cohort of Parkinson’s disease patients, and its knockdown changed mitochondrial morphology (Jansen et al., 2017). Mutations in BLTP3A by contrast are found as a susceptibility locus for systemic lupus erythematosus (SLE), a debilitating autoimmune condition where episodes are often triggered by exposure to UV light. Consistent with the key roles BLTPs play in signalling, membrane homeostasis, and autophagy, mutations or low copy numbers in proteins of this family are also found in cancers (An et al., 2012; Furukawa et al., 2011; Yang et al., 2016; Kang et al., 2009), whereas dominant inheritance of a chromosomal duplication including ATG2B predisposes patients to myeloid malignancies, which may progress to leukemia (Saliba et al., 2015), BLTP3A is upregulated in lung adenocarcinoma (Dan et al., 2021), and BLTP2 is upregulated in several cancers (Song et al., 2006) including breast cancers where it is associated with increased invasiveness and cell metastasis (Banerjee et al., 2024; Zhong et al., 2018).

While this is a rapidly developing field, several recent reviews offer insight into the functional role of this protein family and their effects on membrane traffic and signalling properties in model systems (Levine, 2022; Dziurdzik and Conibear, 2021; Hanna et al., 2023; Vacca et al., 2024; Pandey et al., 2023; Melia and Reinisch, 2022). Taken together, BLTPs facilitate the transfer of lipids from donor membranes—most commonly, but not exclusively, the endoplasmic reticulum (ER)—to target organelles. The target of lipid transfer is dictated by the repertoire of organelle-specific interacting partners associated with each BLTP, which can dynamically vary depending on the availability of these adaptors. This suggests that BLTPs work by the formation of transient, rapidly dissociable membrane contacts, allowing for modulation of membrane composition and functional properties of target organelles on demand.

### Open questions

#### Is lipid transport through RBG proteins directional?

While it is debated whether all members of the BLTP family mediate lipid transfer in one direction only, most of the bridge-like proteins themselves appear to span membrane contact sites in consistent directions, co-ordinated by interactions with adaptors that are specific to the N and C termini of these proteins. This is most clearly borne out in the interactions of the single VPS13 protein in yeast, where N-terminal ER-to-C-terminal organelle contacts are formed by competitive recruitment of the C-terminus to organelle-specific adaptors (Bean et al., 2018). Even there, the list of competing interactors is likely to be further extended, as yeast VPS13 is also detected at junctions between the yeast vacuole and mitochondria(Gonzalez Montoro et al., 2018), suggesting a different N-terminal adaptor, or a tripartite junction including ER. Nevertheless, this suggests that the orientation of the BLTP at a given junction is important. Interestingly, one recent preprint (Hanna et al., 2025) suggested that some BLTPs may re-orient in response to signalling. In the scenario, it is assumed that the change in orientation determines whether the BLTP3A bridge forms between lysosomes and vesicles (at rest) or between the ER and lysosomes (under lysosomal damage conditions), via a rapid change in N and C terminal interacting partners. However, it is still unknown whether the direction (N-terminus to C-terminus) of lipid transfer changes. Previous *in vitro* experiments with the closely related BLTP3B/SHIP164 (Hanna et al., 2022) have demonstrated that recombinant BLTP3B protein is capable of dimerising in a head-to-tail orientation, but it is unclear whether this is a conformation found *in vivo*. If it were, lipids would translocate topologically ‘backward’ over one-half of the junction, and lipid transfer would not be an intrinsic property of the mechanism enabling lipid passage through the RBG hydrophobic groove.

While the example of BLTP3A is the most striking to date, it is apparent that the selection of membranes that the BLTPs transfer to and from is largely determined by the N and C terminal interacting proteins of these family, as the BLTPs do not display strong intrinsic preferences for target junctions in the absence of other factors recruiting them to membranes. As described in yeast, overexpression of proteins that can recruit VPS13 family proteins [the adaptor proteins mcp1 (John Peter et al., 2017), ypt35 (John Peter et al., 2017), and spo71 (Park et al., 2013)] compete (Adlakha et al., 2022; Bean et al., 2018) amongst each other to recruit the single yeast VPS13 protein to different ER-organelle membrane contact sites. This is somewhat distributed over VPS13 paralogues in other species, where each VPS13 has an evolutionarily distinct domain arrangement (Figure 2; Levine, 2022; Leterme et al., 2023). In humans, each of the four paralogues (VPS13A-D) have specific interactors which act together to recruit the family to target membranes (largely via specialised interactions to the VPS13 C-terminus, see Table 2), but even so, each human VPS13 is found at multiple organelle junctions.

Other proteins of the family appear to be less specialised, and it is unclear whether the distinction between the donor and acceptor ends of the protein (and therefore, the direction of lipid transport over these bridges), or the conditions for RBG proteins to be recruited to organelle junctions, is as highly regulated as it appears to be for the ATG2/VPS13 grouping when expressed at endogenous levels.

In some of these proteins, the selection of a ‘donor’ end of the protein may be fixed: BLTP1 has recently been identified with its N-terminal transmembrane helix in complex with two proteins (spigot/C1orf43, an ER-resident protein that cups the N-terminal chorein-like domain, and intake, a nematode-specific helical transmembrane protein which contacts both BLTP1(*C. elegans* LPD-3) and spigot) (Kang et al., 2024a), while BLTP2 sports its own N-terminal transmembrane helix. At the C-terminal, BLTP1 has a C-terminal amphiphilic helix and polybasic patch, which would be expected to interact with charged phosphoinositide lipid and perhaps dock to membranes (Wang et al., 2022), but would be far easier to dynamically dissolve and remodel (to perhaps select a new target membrane) than the interactions supported by the N-terminus. It is not clear whether the transport of its lipid cargo is in fact uni-directional (even if the orientation of the protein is fixed) as BLTP1 is suspected of being capable of transporting lipids between ER and plasma membrane bidirectionally (Toulmay et al., 2022; John Peter et al., 2022a) at its endogenous levels of expression.

Nevertheless, it would appear that at least some members of the BLTP family have specialisations that may favour unidirectional transport, particularly the VPS13/ATG2 grouping. In the case of VPS13 family proteins, donor/acceptor membranes are also largely determined by protein–protein interactions at the C-terminus of the protein (see Table 2). Interestingly, in at least some subcellular organelles, ATG2 and VPS13 proteins are partially redundant (Lei et al., 2022; Dabrowski et al., 2023), suggesting that despite refined mechanisms for targeting these proteins, which become relevant in human disease, the fundamental property of bulk lipid transfer is the most important facet of their function.

#### How selective are RBG transporters for specific cargo lipids?

Generally, evidence shows that the chorein domains of BLTPs can bind and solubilise the major membrane phospholipids [phosphatidylethanolamine (PE), phosphatidylcholine (PC), phosphatidic acid (PA), phosphatidylserine (PS), phosphatidylinositol (PI), and phosphatidylglycerol (PG)] and are a poor selector for cholesterol (reviewed here; Hamai and Drin, 2024). However, it does not appear that the chorein domains are particularly selective for their cargoes, which appear to be representative of the major glycerophospholipid classes present in donor membranes. Studies looking at the affinity or transport of lipids show that in recombinant systems, BLTPs can transport PE, PC, PA, and PS and to some extent PI (Kumar et al., 2018; Hancock-Cerutti et al., 2022; Valverde et al., 2019), as well as fluorescent nitrobenzoxadiazole (NBD)-labelled lipids. Deletion of Atg2 in *S. cerevisiae* stops R18 (octadecyl rhodamine B) transfer (Hirata et al., 2017). R-18 is a single chain C-18 lipid with a hydrophilic fluorescent ‘headgroup’, illustrating the lack of headgroup selectivity of BLTPs. In chimeric assays, the chorein domains of ATG2 and VPS13 can be swapped (Osawa et al., 2019) and still retain function.

As the research currently stands, selectivity for lipids is more likely to be driven by the recruitment of the complex to the appropriate junction, and regulation by the properties of the two membranes driving a transport gradient. Assays in reconstituted systems show that BLTP3B rapidly retrieves a packet of lipid from the donor liposomes and then remains stable if there is no acceptor liposome to receive transferred lipid, suggesting that donor lipid is moving down an internal gradient within the RBG tube (Hanna et al., 2022), which drives extraction from the donor.

**Figure 3:**
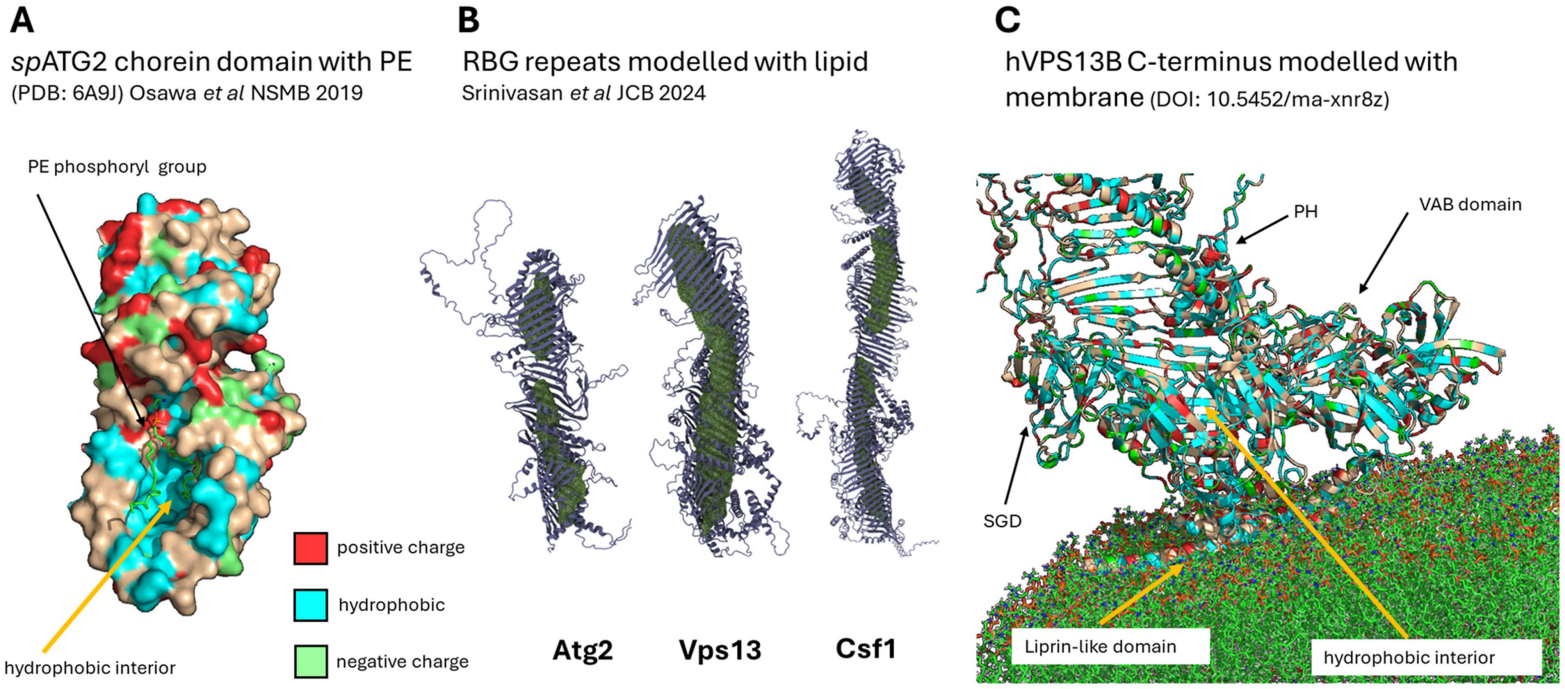
Lipid interaction with BLTPs. **(A)** X-ray crystallography of a single PE molecule with *S. pombe* ATG2 chorein domain (PDB:6A9J) showing acyl chain docking to the hydrophobic interior of the chorein domain, and phosphoryl group of PE close to a positive charge at the chorein domain mouth. **(B)** Computational model of lipid docking to the open ends of RBG repeats shows that there are discontinuous ‘bottlenecks’ where lipid is not docked in the interior of some RBGs. Image reproduced from Srinivasan et al. (2024), under the terms of the Creative Commons Attribution–NonCommercial–ShareAlike 4.0 International license. **(C)** Model of the C-terminal liprin-like domain of VPS13B interacting with model membranes [Modelarchive ma-xnr8z; (Dall’Armellina et al., 2023)], where the liprin-like domain docks on the target membrane, disturbs the cytosolic leaflet, and provides a continuous track to the hydrophobic interior of the RBG for lipid deposition on the target membrane.

X-ray crystallography resolved the *S. pombe* ATG2 chorein domain in complex with PE (Osawa et al., 2019), where the majority of the interactions were with the acyl chains of the lipid, with what appeared to be a relatively loose selectivity for acyl chain length and little interaction with the lipid headgroup except a weak interaction with the PE phosphoryl group stabilised by an arginine residue in the chorein domain (see Figure 3A). It is also possible that some lipids are incorporated into the flow of lipids into chorein domains as ‘passengers’ and are not necessarily selected for. Molecular dynamics (Wang Y. et al., 2024) models favour an entry mechanism of spontaneous absorption of PC by the hydrophobic chorein domain interior, when the acyl chains of the absorbed lipid have been exposed to the cytosol by membrane bending (e.g., a highly curved membrane modelled in this case by a 1,2-dioleoyl-sn-glycero-3-phosphocholine (DOPC) micelle). This process appears to involve a number of conformational steps in the chorein domain: First, one of the two DOPC acyl chains spontaneously inserts into the hydrophobic chorein domain cavity while the charged headgroup interacts with a charge at the ‘mouth’ of the chorein domain scoop; second, the charged headgroup interacts with other slightly deeper positively charged residues to flip the polar head of PC into the cavity; finally, the second acyl chain trails behind and the lipid is transferred onwards, dragging a new acyl chain from the next lipid to enter behind it, potentially generating a continuous stream of oriented lipid. It is speculated that the energy required to drive these conformational changes in the chorein domain, particularly that needed to untangle one acyl chain from its hydrophobic association with other lipids in the micelle, may be supplied by interacting proteins that lower this energy barrier.

#### Are lipids driven through BLTPs by gradients between donor and acceptor?

Current thinking suggests that BLTPs exploit membrane gradients to transfer bulk membrane phospholipids. Unless a gradient is built by the expenditure of energy (e.g., enzymatic activity), BLTPs would exploit passive gradients between membranes, as well as the properties of the transported lipids themselves. Some gradients are easily generated by local phenomena (e.g., placing the BLTP very close to a lipid synthase on the donor membrane (Lees and Reinisch, 2020) or a lipid hydrolase at the acceptor membrane, thus causing local depletion), or by deformation of the target membrane which may cause lipids to sort away from their site of arrival on a membrane. Liposome curvature has been shown to drive BLTP-mediated lipid transfer *in vitro* (Chowdhury et al., 2018), most likely because lipids are already partially exposed to the cytosol as the bending angle increases and thus are more easily solubilised by the chorein domain, and/or added more easily to the acceptor membrane as exposure of acyl chains on the bent target membrane favours the addition of further lipids.

The disturbance of acyl chains on the donor membrane is likely to facilitate lipid transfer. In models of the interaction of the liprin-like C terminal domain of VPS13 family proteins interacting with membranes, the liprin-like domain disturbs the cytosolic leaflet of the acceptor membrane bilayer, exposing the hydrophobic lipid chains to the ‘incoming’ lipid being donated by the BLTP, thus likely increasing the efficiency of their incorporation in the target membrane. Interestingly, several missense mutations of this liprin-like domain cause disease in human VPS13s, suggesting their relevance to the lipid transfer process (Dall’Armellina et al., 2023). Recent structural studies (Wang Y. et al., 2024) showed that in human ATG2A, this domain is extremely flexible with respect to its position relative to the RBG tube and assists in feeding lipids to the ATG9A scramblase in the target membrane.

Consistent with this mechanism being exploited by BLTPs *in vivo*, examination of subcellular organisation places lipid transfer via ATG2 to the very edge of the growing omegasome (Chowdhury et al., 2018), and BLTP2 is also found at the tips of tubular endosomes defined by Rab8 and Rab10 (Parolek and Burd, 2024), both where the curvature is highest.

Other properties, such as lipid packing, can also be sensed and/or rectified via BLTP-mediated transfer, suggesting that gradients of lipid composition or fluidity also drive BLTP function. This is illustrated in the case of the role of *C. elegans* lpd-3/BLTP1 countering the effect of cold stress on membrane fluidity (Wang et al., 2022), which is discussed below.

An important consideration is that the action of BLTPs is to drive bulk lipids to the cytosolic leaflet of a membrane bilayer, which then may or may not be redistributed by a scramblase. Eukaryotic membranes favour a less fluid, more tightly packed outer membrane leaflet, while the cytosolic face of the membrane bilayer (the target of BLTP-mediated transfer) is more loosely packed, thus allowing spontaneous membrane deformation (Bogdanov, 2023), and consequent segregating and sorting of membrane subdomains, which may influence a number of properties of the acceptor membrane. While the ER, the most common source of donor lipid for BLTP-mediated transfer, is both enriched in lipid synthases and relatively symmetric across inner and outer membrane leaflets, the acceptor organelles are successively more and more cholesterol-enriched (Maxfield and van Meer, 2010) and asymmetric [the outer leaflet being more rigid and the cytosolic leaflets of most organelles and both leaflets of the ER harbouring more flexible unsaturated lipids (Dingjan and Futerman, 2021)]. Thus, it is conceivable that the deposition of lipids will allow significant changes to membrane signalling due to localised changes in membrane properties.

How the nature of the donor membrane composition may impact proteins such as BLTP1, which appears to transfer lipids via its N-terminal helix and partner transmembrane protein C1orf43 (Kang et al., 2024a) from the PS-rich luminal leaflet of the ER at to the similarly PS-enriched cytosolic leaflet of target membranes (Kobayashi and Menon, 2018), while most other ER-resident lipids are symmetrically distributed across bilayers, is yet to be explored. It is intriguing, however, that both BLTP1 and C1orf43 are required for phagocytosis of *L. pneumophila* (Jeng et al., 2019) by macrophages and for phagocytosis of neuronal debris (Kang et al., 2024a), suggesting that continuous access to the ER lumen is necessary for function. Similarly, yeast Csf1/BLTP1 immunoprecipitated the ER lumenal enzyme Mdc4 (Toulmay et al., 2022), which depletes PE for the synthesis of GPI anchors. This may suggest that local depletion of PE in the lumenal bilayer is relevant to BLTP1 function.

#### Can lipid transport through the RBG bridge be regulated?

Several models have been made of lipid interactions with members of the BLTP family, which all favour a continuous stream or bolus of lipid passing through the RBG portion of BLTPs (Li et al., 2020), following the hydrophobic track of residues down the centre of the RBG as predicted by mutagenesis studies (Valverde et al., 2019; Tan and Finkel, 2022). It is unclear whether, having entered the RBG ‘tube’, lipids are subject to any flow regulation as they pass from one side of the BLTP bridge to the other, but some indications exist that some kind of flow regulation or management may take place.

As lipids traverse the RBG repeats, modelling and cryo-electron microscopy find that BLTPs are suited to the co-ordinated deposition of multiple lipids at a time, which allows the lipids to orient and group themselves, thus minimising the energy needed to transfer lipids from one membrane to another. Recent cryo-electron microscopy studies (Kang et al., 2024a) and assays with NBD-PE in reconstituted systems (Hanna et al., 2022) show multiple lipids at a time are docked in the RBG groove.

Recent and elegant molecular dynamic (MD) simulations of lipid docking on open membrane-less models of the RBG portion of BLTPs (Srinivasan et al., 2024), shown in Figure 3B, show no intrinsic preference in terms of direction of entry for the open RBG repeats of ATG2 in MD simulations of free lipids in solution with BLTPs. Simulating sequential docking of multiple free lipids in yeast Atg2, Vps13, and Csf1/BLTP1, the authors show that these proteins can carry in the RBG repeat ‘tube’ at least 15, 49, and 53 phospholipids, respectively, where the charged lipid headgroup faces the solvent. Interestingly, this modelling also showed that the docked lipids were not equally distributed down the length of the RBG tube, instead finding bottlenecks in the hydrophobic track of Atg2 and Csf1/BLTP1 where lipids did not preferentially reside, which may provide an opportunity for transport down the length of the RBG tube to be regulated. These models, where only the open RBG tube is considered, would favour the bi-directional transport of lipids via BLTPs as the free lipids entered from both N and C terminal directions of the modelled open tubes. These bottlenecks in RBG repeats were also found in cryo-EM structures of ATG2A (Wang Y. et al., 2024), suggesting that the passage of lipids through BLTPs is not uninterrupted, and may require changes in conformation to allow passage of lipids. Several possible methods of regulation may exist that would modulate the diameter of the RBG tube during lipid transport, such as locally twisting or compressing RBG repeats, either by the many conserved flexible repeats and motifs that are scattered along the length of RBG proteins (Levine, 2022; Dall’Armellina et al., 2023) or by physical changes in the distance between membranes which may act as a kind of pump to drive lipids across the hydrophobic path.

Other models of BLTP family interaction with lipid in RBG bridges agree with a model of interrupted, and therefore regulatable, lipid transfer. A recent preprint (Kang et al., 2024a) showed that, in cryo-EM studies of *C. elegans* lpd-3/BLTP1 extracted with its native complex and cargo lipids, this protein was intimately twined via BLTP1’s N-terminal transmembrane helix and its chorein domain with two ER transmembrane proteins (intake, a *C. elegans* specific gene, and C1orf43/spigot) which allowed a continuous hydrophobic path from the ER lumen to the interior of the BLTP1 RBG repeats, suggesting BLTP1 is continuously anchored to its donor membrane and in contact with transportable lipid. In this study, Kang et al. were able to reveal some very interesting features of the interaction of phospholipid with RBG repeats. First, they noted that the distribution of phospholipids along the RBGs resolved in their structure was not homogenous: The narrower ~25 Å RBG tubes at the N-terminus of BLTP1 harboured a single co-ordinated row of phospholipids, whereas the broader (~40 Å) RBG repeats towards the middle of the protein were associated with 2–5 rows of lipid within the RBG repeat. Most interestingly, while the hydrophobic tails of these lipids faced the hydrophobic interior, the phospholipid headgroup was oriented towards the water in the half-open RBG repeat, by the presence of alternating acid and basic residues, which coordinated the lipids in rows. The more ionisable residues per RBG repeat, the more rows of lipids could be co-ordinated, such that in the wider RBG repeats, lipids were packed at a similar density to one leaflet of a membrane bilayer. The authors speculate that this allows the interior of the RBG repeat to conduct oriented clusters of lipids as if they were small patches of a membrane leaflet. The continuous anchoring of BLTP1 with membrane integral proteins at its N-terminus, and the accommodation of more lipids at the centre of the bridge than at its beginning would likely drive a gradient of movement away from the donor membrane. Interestingly, this was also observed in ATG2A, where, except for a bottleneck at the N terminal region, the hydrophobic track broadened along RBGs until the last segment, which contacts the acceptor membrane. This segment was unresolved in these structures, suggesting an internally generated lipid transfer gradient from the N-terminal to the C-terminal of the BLTP (Wang Y. et al., 2024).

Unfortunately, it was not possible to solve the structure of the entire BLTP1 complex *ex vivo* at the resolution needed to identify docked lipids over the entire length of the membrane bridge, so the nature of the interaction of BLTP1 with the plasma membrane was not observed in this system. Computational models of BLTP1 structure favour a widening of RBG repeats from the N- to C-terminus, until reaching a narrow neck at the C-terminal region of the protein (Wang et al., 2022). However, BLTP1 (Toulmay et al., 2022; John Peter et al., 2022a) like SHIP164/BLTP3B (Hanna et al., 2022) is of the subgroup of BLTPs for which bidirectional lipid transport is suspected, and this correlates with very little specialisation of the C terminal acceptor end of the BLTP bridge.

There are arguments, however, that the more specialised members of the BLTP family (ATG2, VPS13) for which the evidence for unidirectional lipid transport is stronger, may have adopted features that make lipid transport favour the N-terminal to C-terminal direction. Our own study modelling interactions of the specialised N- and C-terminal domains of VPS13A-D with donor and acceptor membranes (Dall’Armellina et al., 2023) showed that the liprin-like helical bundle, which is found in ATG2/VPS13 proteins, allows a narrowing of the extended RBG repeats until a single helix with a hydrophobic face covers the last distance between the RBG tube and where the liprin-like domain disturbs the cytosolic face of the target membrane (Figure 3C). We speculate that these features reduce the ability of solubilised lipids to ‘crawl’ back up the RBG tube and thus impose a direction of transport, in addition to the liprin-like domain facilitating lipid admixture by disturbing the acceptor membrane cytosolic leaflet.

Based on our modelling on VPS13s, along with work from others since, we propose two models to regulate the passage of lipids through RBG bridges: (1) a spring-like action of compressing and expanding RBG repeats (which potentially may be regulated by interaction with the many conserved cytosolic loops and motifs down the length of the BLTP proteins), and (2) a transient interaction model, where VPS13 RBGs are rigid, and lipids fall down the gradient between donor and acceptor membranes. In this model, the N-terminal chorein domain is weakly associated with donor membranes via long, flexible tethers to proteins such as VAPA/B, allowing transient ‘gulps’ of donor lipids. This could well be similar in other BLTPs, as current evidence suggests that BLTPs are strongly associated with either their donor (BLTP1-2) or their acceptor (VPS13) membranes, but not both—allowing transient interactions with one side of the intraorganelle bridge to drive pulses of lipid down the RBG tube.

#### How fast are BLTPs?

The presence of BLTPs, despite their apparent lack of selectivity, also provides some advantages when dynamically manipulating membrane properties, as bulk transfer allows movement of lipids between organelle membranes on the scale of minutes (Wong et al., 2019; Reinisch and Prinz, 2021) thus allowing fast and presumably local response to changes in membranes: This is particularly seen by the action of VPS13C (Schrӧder et al., 2024; Wang X. et al., 2024) BLTP3A(Hanna et al., 2025) and ATG2 (Tan and Finkel, 2022) being recruited to damaged lysosomes for the process of lysosomal repair—a process that is accomplished on the scale of minutes and heavily involves a variety of other LTPs outside of the BLTP family.

*In vitro* studies using Förster resonance energy transfer (FRET)-based assays in liposomes have measured the transfer of a 2% mix of NBD-PE from donor liposomes at a surprisingly low rate of ~0.017 s-1 per molecule of the BLTP (Maeda et al., 2019). However, subsequent reanalysis of the composition of those liposomes, along with the fact that BLTPs unselectively transport phospholipids regardless of labelling, suggests that the maximal lipid flux per BLTP molecule in that same assay is 50 times higher, at 0.85 molecules s-1, (Hamai and Drin, 2024). This occurs even when there is not necessarily a very strong gradient between donor and acceptor liposomes, limiting the drive to traverse the BLTP. Assays where yeast Atg2-Atg18 are coupled with Atg9 scramblase show a doubling in the efficiency of *in vitro* NBD-PE transfer between liposomes (regardless if Atg9 was interacting with Atg2 on donor or acceptor liposomes), suggesting that transfer rates are dependent on whether both the inner and outer leaflets of membranes are accessible to lipids donated by BLTP complexes (Chumpen Ramirez et al., 2023).

*In vivo* measurements, however, put the lipid transfer rate much higher: ~200 lipids per second per molecule of Atg2 in yeast (Dabrowski et al., 2023). In all, this suggests that the rate at which lipids traverse the BLTP will be very much context-dependent, responding to local membrane geometry, membrane composition, and the role of BLTP-associated proteins in processing the lipids that are passing through.

#### What effect does BLTP-mediated lipid transport have on target membrane geometry?

Insights into the consequences of lipid transfer mediated by BLTPs have been gained through structure–function analyses and knockout studies across various organisms and cell types. Collectively, these findings suggest that the effects of BLTP-mediated lipid transfer depend on both the specific lipid being deposited and the manner in which the donated lipid is incorporated with the acceptor membrane. This process influences the geometry and biophysical properties of the target organelle, ultimately modulating cell signalling pathways and membrane sorting.

A key factor in determining how acceptor membranes are altered lies in the presence of accessory complex components associated with BLTPs. These proteins often function as part of larger macromolecular assemblies, interacting with accessory proteins such as Rabs and adaptor proteins or associating with membrane scramblases, which modulate the impact of the transferred lipid on the target membrane. The specific targeting and interaction partners at each BLTP-mediated intraorganellar contact appear to dictate the functional outcome of BLTP activity at a given membrane junction.

BLTPs are thought to deposit bulk membrane lipids onto their target membrane, but the context and method of deposition have different effects on the membrane properties of the target organelle. Experiments show BLTPs can drive membrane expansion in the case of VPS13/ATG2 proteins (Chowdhury et al., 2018; Da Costa et al., 2020) favouring models where these more specialised BLTPs do in fact drive unidirectional transport. Other members of the family such as BLTP1-2 have less specialisation at their C-termini but nevertheless drive changes in membrane properties such as membrane fluidity and consequent signalling.

One key distinction between the actions of BLTPs in their different cellular contexts is the presence or otherwise of associated scramblases as this imposes a geometry as to how the transported lipid will be distributed on the acceptor membrane. These transmembrane proteins facilitate donated lipids accessing the lumenal leaflet of the membrane bilayer, which has the effect of equilibrating the donated lipid across both bilayers of the membrane, which allows expansion of acceptor membranes without much bending. This is best understood for the VPS13/ATG2 grouping of BLTPs. It was noted that VPS13A associates with the scramblase XK (Ryoden et al., 2022) and shares a common disease phenotype (Park and Neiman, 2020) where PS exposure and PC internalisation of the outer face of the plasma membrane are compromised. In yeast, VPS13 forms a complex with the yeast-specific MCP1 protein, which, despite no sequence conservation with XK, also has scramblase activity (Adlakha et al., 2022). This is also the case for the similar ATG2 proteins that interact with the lipid scramblases ATG9a, TMEM41B, and VMP1(Ghanbarpour et al., 2021; Maeda et al., 2020). Cryo-EM suggests that human ATG2A directly feeds lipid to the lateral pores found in a single ATG9 unit in a target membrane, funnelling transferred lipid to the central ATG9A scramblase pore formed by ATG9A trimers (Wang Y. et al., 2024), and thus to the equilibrated membrane bilayer.

In these cases, the equilibration of the inner and outer leaflets of the target organelle will also drive transfer gradients by spreading the donated lipid over both the inner and outer leaflets of the membrane bilayer, by helping to maintain a concentration gradient between the donor membrane and the acceptor membrane cytosolic leaflet. This method of lipid deposition is supported by the established role of scramblase-associated ATG2 in depositing lipids for phagophore formation (Maeda et al., 2019; Chowdhury et al., 2018; Valverde et al., 2019; Osawa et al., 2019). Interestingly, viral replication of tomato bushy stunt virus in yeast and plants requires both Atg2 and its associated scramblase Atg9 (Kang et al., 2024b) showing the importance of this mechanism to ATG2 function.

In proteins such as BLTP3B/SHIP164, which are not known thus far to form complexes with a scramblase, overexpression of the BLTP and a targeting protein (in this case its interactor, syntaxin 6) leads to excessive tubulation of endosomes, possibly driven by the asymmetric delivery of lipids to the outer leaflet of the acceptor membrane, which, if not equilibrated, would need to deform and bend to accommodate the excess lipid (Otto et al., 2010). This change in membrane geometry leads to changes in the trafficking of endosomal cargoes such as cation-independent mannose 6 phosphate receptor and transferrin receptor. This also appears to be the case for the VPS13 family of proteins when these proteins are not interacting with scramblase, where VPS13-mediated transport then favours membrane distortion: Yeast VPS13 directs lipid to lysosomes to accomplish ESCRT-mediated inward budding and membrane sorting to form ILVs (Suzuki et al., 2024) or VPS13B allows the formation of extended tubular ERGIC to accommodate the trafficking and secretion of long extracellular proteins such as procollagen (Du et al., 2024). Similarly, *Drosophila* BLTP1, apart from other roles in neurons, also has a role in astrocytes, where its depletion interferes with the formation of phagosomes for clearance of neuronal debris(Kang et al., 2024a; Kang et al., 2023).

#### Do BLTPs change the signalling properties of membranes?

Although BLTPs transport lipids that are abundant in most membranes, the donor and acceptor membranes connected by BLTP bridges exhibit distinct characteristics, such as the different phosphatidylinositol phosphates (PIPs) that define organelles, and varying levels of cholesterol, which does not appear to be transported as cargo. They also differ in lipid composition (e.g., PC/PE/PS ratio), lipid saturation levels, and the level of asymmetry between the cytosolic and lumenal/extracellular leaflets of the bilayer [reviewed here (Kobayashi and Menon, 2018; Van Meer et al., 2008)].

The rapid admixture of lipids from other organellar membranes, such as the relatively unsaturated lipids found in fluid ER, the site of synthesis for most of the lipids known to be transported via BLTPs, is likely to change the properties of both donor and acceptor membranes. These changes may be to the geometry (e.g., cone-shaped lipids such as PE favour membrane bending, and unsaturated lipids favour membrane flexibility), or the capacity of the membrane to form ordered domains for signalling [as, e.g., is found at ER-membrane contacts (King et al., 2020)]. This has been proposed for BLTP2, which drives lipids from the ER to the plasma membrane to donate lipids to macropinosomes and tubular endosomes as they fuse to the plasma membrane (Dai et al., 2025). The absence of BLTP2 in this context leads to the accumulation of vacuolar structures that are contiguous with the plasma membrane and open to extracellular space, showing the importance of lipid deposition and changed local membrane properties via these proteins to assist membrane traffic, membrane deformation, and consequent signalling.

Nevertheless, there is a degree of subtlety as to how each BLTP is able to change membrane properties. In systems where some level of functional redundancy might be anticipated between members of the BLTP family such as BLTP2 and BLTP1—which both mediate transfer between ER and the plasma membrane—certain functional roles are specialised. These roles may be influenced by specific protein interactions with these BLTPs, as well as by the physical context of their membrane junctions and the local curvature induced by lipid transfer. Both BLTP1 and BLTP2 have important roles in homeoviscous adaptation (HVA), that is, maintaining membrane sorting and signalling properties under cold stress (Ernst et al., 2016). This network of adaptations is large, contextually dependent, and involves many different potential lipid modifications, or selections for properties [e.g., acyl chain length or saturation (Ernst et al., 2016; Ballweg et al., 2020)]. *C. elegans* LPD-3/BLTP1 is responsible for mitigating membrane rigidification (such as cold stress), by depleting PC from the ER membrane and blocking compensatory upregulation FAT-7 fatty acid desaturase, which uses an alternate path to increase membrane fluidity (by generating unsaturated acyl chain on resident lipids). Interestingly, the BLTP1 function can be bypassed by the addition of unsaturated phospholipids (lecithins) to the plasma membrane (Wang et al., 2022; Pandey et al., 2023). In yeast, CSF1/BLTP1 mutations cause a cold-sensitive phenotype (Tokai et al., 2000), which manifests as a lack of adaptive response (generation of shorter acyl chain lipids and increased acyl chain unsaturation), which could be in part reverted by treatment with the unsaturated lipid oleic acid (John Peter et al., 2022b).

BLTP2 regulates membrane fluidity by increasing PE levels at PM, facilitating cancer growth (Banerjee et al., 2024). Its yeast orthologue, Fmp27, exhibits a cold-dependent growth phenotype, resolved by supplementation with propanolamine, a lipid similar to PE that cannot be converted to PC, suggesting a specific requirement for BLTP2-delivered PE at these sites. While wild-type yeast acclimatise to cold stress by raising the plasma membrane PE:PC ratio, yeast lacking Fmp27/BLTP2 fail to increase plasma membrane PE (Banerjee et al., 2024). The authors also showed that a shift to cold temperature provoked a change in lipid unsaturation in both cell lines, which was abolished by the presence of abundant ethanolamine, a precursor to PE, indicating redundant pathways maintaining membrane fluidity in HVA (Banerjee et al., 2024).

PE is also a precursor of GPI anchors, and while mutation of yeast Fmp27/BLTP2 does not appreciably affect the synthesis of GPI anchors (Banerjee et al., 2024), yeast Csf1/BLTP1 mutants have disturbed GPI anchor biosynthesis (Toulmay et al., 2022). This highlights that although broadly similar, specificity is present between the effects of lipid transport by BLTPs and thus must be conveyed by either location or admixture of lipids.

However, the downstream consequences of altering membrane properties, even by manipulation of the highly abundant phospholipids that BLTPs are known to transfer, are considerable. Changing the balance of PE to PI, where PE is a curvature promotor and PI, because of hydrogen bonding between headgroups, favours uncurved membrane (Klose et al., 2012), induces membrane bending, lipid segregation, and consequent organisation of signalling complexes which affect minor membrane components and signalling lipids such as phosphoinositide lipids (PIPs). This is best illustrated in the downstream consequences of BLTP1 mutation in various model systems. In *C. elegans* and mouse fibroblasts, BLTP1 mutants show defects in the positioning and abundance of the low-abundance PIP lipid PI(3,4,5)P_3,_ as well as disorganised actin polymerisation (Wang et al., 2022; Khuong et al., 2010). In contrast, in *Drosophila melanogaster,* BLTP1 loss affects the plasma membrane positioning and abundance of PI(4,5)P_2_, leading to defects in synaptic vesicle cycling (Verstreken et al., 2009; Khuong et al., 2010). It also appears that loss of this pathway triggers other strong signalling across membranes, leading to an insulin-dependent hyperactivation of mTOR signalling and consequent reduction in lifespan for LPD-3/BLTP1 mutants in *C. elegans*, which could be rescued by lecithin (Pandey et al., 2024), whereas insulin secretion is compromised in *Drosophila* Hobbit/BLTP2 mutants (Neuman and Bashirullah, 2018). A change in PIP signalling has also been observed in target membranes of cells depleted of VPS13 family proteins (Park et al., 2015), suggesting common effects of bulk phospholipid transfer by BLTPs on membrane signalling properties *via* PIP lipids.

It is unclear whether these effects on PIP lipid signalling are due to a direct defect in PIP lipid transfer *via* the RBG bridge, or the deficit in PIP lipids stems from downstream consequences of changes to membrane fluidity and therefore recruitment of specific PIP kinases and phosphatases, which preferentially segregate to lipid microdomains defined by acyl chain composition (Wang and Richards, 2012; Wenk et al., 2003).

## Conclusion

Recent studies have identified several common characteristics of BLTPs: (1) a loose association with either donor or acceptor membranes; (2) the presence of internal gradients that promote lipid accumulation in regions with wider RBG repeats; (3) lipid transfer “bottlenecks,” which may serve as regulatory points for lipid transfer; and (4) effects on PIP lipid signalling, likely secondary to the transfer of major lipid constituents such as phosphatidylcholine (PC) and phosphatidylethanolamine (PE). Whether bi-directional or unidirectional, the effects of lipid transfer appear to be driven by both the gradient of phospholipids between donor and acceptor membranes and association with other membrane proteins which drive lipids to equilibrate or not over associated membrane bilayers. The presence of these accessory complex members imposes specific membrane geometries and lipid sorting patterns, with significant implications for protein distribution and membrane signalling. Overall, the studies conducted so far suggest that from the simplest of principles (a scoop that can load lipids and a tube that can conduct it in co-ordinated patches), highly specific, context-dependent changes can be made to membrane function at all levels of membrane sorting and signalling, underpinning neuronal and organismal function. Our growing understanding of such an ancient, seemingly simple, group of lipid transfer proteins suggests that BLTP-mediated regulation of phospholipid gradients can orchestrate extensive and surprisingly sophisticated changes in cellular signalling and interactions with the extracellular environment.

## ‘Glossary

ATG2: Autophagy-related 2
BLTP: Bridge-like lipid transporter
CASM: Conjugation of Atg8 to single membranes
ChAc: Chorea-acanthocytosis
ER: Endoplasmic reticulum
ERGIC: ER-Golgi intermediate compartment
ESCRT: Endosomal sorting complex required for transport
FFAT: Two phenylalanines in an acidic tract
GTPase: Guanosine triphosphatase
ILV: Intraluminal vesicle
LC3: Microtubule-associated proteins 1A/1B light chain 3
LIR: LC3-interacting region
MLD: Mitochondrial-associated membrane localisation domain
mTOR: Mechanistic target of rapamycin
NBD: Nitrobenzoxadiazole (fluorescent lipid label)
PA: Phosphatidic acid
PC: Phosphatidylcholine
PE: Phosphatidylethanolamine
PG: Phosphatidylglycerol
PH: Pleckstrin homology
PI: Phosphatidylinositol
PM: Plasma membrane
PS: Phosphatidylserine
RBG: Repeating beta groove
RNA: Ribonucleic acid
RXP: Arginine-X-Proline motif
SH3: Src homology 3
SNARE: Soluble NSF attachment protein receptor
TSG101: Tumour susceptibility gene 101
UHRF1BP1: Ubiquitin-like with PHD and RING finger domains 1 binding protein 1
UHRF1BP1L: UHRF1BP1-like protein
VAB: VPS13 adaptor-binding domain
VAMP7: Vesicle-associated membrane protein 7
VAPA/B: Vesicle-associated membrane protein-associated protein A/B
VMP1: Vacuole membrane protein 1
VPS13: Vacuolar protein sorting 13
WD40: Tryptophan (W) aspartate (D) motif of 40 amino acids
WIPI: WD repeat domain phosphoinositide-interacting protein
WWE: Trp Trp Glu motif domain
XK: Kell blood group precursor
